# The association between consumption of red and processed meats with metabolic syndrome and its components in obese and overweight women: a cross-sectional study

**DOI:** 10.1186/s12905-023-02862-7

**Published:** 2024-02-06

**Authors:** Atousa Zandvakili, Farideh Shiraseb, Dorsa Hosseininasab, Yasaman Aali, Raul D. Santos, Khadijeh Mirzaei

**Affiliations:** 1 Department of Nutrition, School of Health and nutrition, Yasuj University of Medical Sciences, Yasuj, Iran; 2 Department of Community Nutrition, School of Nutritional Sciences and Dietetics, Tehran University of Medical Sciences (TUMS), Tehran, Iran; 3 Department of Nutrition, Science and Research Branch, Islamic Azad University, Tehran, Iran; 4 Heart Institute (InCor) University of Sao Paulo, Medical School Hospital, Sao Paulo, Brazil

**Keywords:** Metabolic syndrome, Obesity, Processed meat, Red meat, Women

## Abstract

**Objectives:**

Previous studies have shown a relation between the consumption of different types of meats and chronic disorders. This study aims to investigate the association between red and processed meat intake with metabolic syndrome (MetS) and its components in healthy obese and overweight women.

**Methods:**

This cross-sectional study was conducted on Iranian women. The dietary assessment and body composition were measured by a validated food frequency questionnaire (FFQ) and bioelectrical impedance analysis, respectively. Blood samples were collected by standard protocols.

**Results:**

A total of 231 women (mean age 36.47 ± 8.44 years) were included in the current study. After controlling for potential confounders, there was a marginally significant associations between higher intake of processed meat with the MetS (OR:1.01, 95% CI: 0.94,2.94, P:0.06) and high serum triglycerides (TG) (OR:1.27, 95% CI: 0.94,2.98, P:0.07). There was a significant associations between high intake of red meats with lower odds of higher waist circumference (WC) (OR:0.31, 95% CI: 0.10,0.97, P:0.04). Also, there was a significant associations were found between high intake of processed meats with greater odds of having lower high-density lipoprotein cholesterol (HDL-c) (OR:0.64, 95% CI: 0.30,0.95, P:0.03).

**Conclusions:**

The current study suggests that higher intakes of processed meat may be associated with the MetS in Iranian women with excess body weight, while this was not the case for red meat. More studies however are necessary in different communities to draw definitive conclusions.

## Introduction

The prevalence of chronic diseases such as the metabolic syndrome (MetS) is increasing and this is becoming an urgent global health concern [1]. This disorder is characterized by the association of central obesity, hypertriglyceridemia, hyperglycemia, reduced blood high-density lipoprotein cholesterol (HDL-c), and hypertension [2, 3]. According to epidemiological studies, the global MetS epidemic rate was estimated at 20 to 25% [4]. In the Farmanfarma’s study one-third of Iranian adults are suffering from MetS. Interestingly, the prevalence of MetS is about 10% higher in women than in men [5]. Many etiological factors are attributed to MetS, including obesity [6], alcoholism [7], genetics [6], smoking [7], and diet [6].

Indeed some foods in diet including red meat, have been associated with components of the MetS and many studies have focused on that since this may be a major modifiable factor [2, 8, 9]. Various components in meat such as saturated fatty acids (SFAs) may cause metabolic disorders [10]. Also, iron in red meat can play a role in inducing oxidative stress and subsequent insulin resistance (IR) [11]. In addition, nitrites, nitrates and sodium used as additives in processed meats are considered as risk factors for diabetes and hypertension [12]. Some studies have reported a positive association between meat consumption, especially red and processed meat with high blood pressure (BP), abdominal obesity, and diabetes, which are components of MetS [3, 7, 13]. In a meta-analysis of observational studies by Kim et al., higher consumption of red and processed meats were associated with 33 and 35% increased risk of developing the MetS [3].

According to an explanation and despite the existing controversies regarding the association between consumption of red and processed meats and MetS needed. In addition, this issue has not been investigated sufficiently in Iran, in obese and overweight women, so with a view more comprehensively, the consumption of red and processed meats was examined in the current study.

## Methods

### Study population

In this cross sectional study 231 healthy overweight and obese women aged 18–48 years old were selected from the Community Health Centers of Tehran University of Medical Sciences (TUMS) in Theran, Iran. A total sample size of participants was determined by the this formula (([(Z1 − α + Z1 − β) × √1 − r^2^]/r)2 + ^2^), β:0.95, α:0.05, with 95% confidence and 80% power, and r:0.25) [14]. Among the 25 comprehensive health centers that were covered by Tehran University of Medical Sciences, 20 centers were randomly selected according to the sample size. The participants were included in the study based on the entry and exit criteria and by multi-stage random sampling method.. The inclusion criteria included consent to participate in the study, the health conditionof participants, female gender, BMI between 25 and 40 kg/m^2^. Participants who had the following conditions were excluded: consumption of alcohol, smoking, participating in a weight loss program or taking weight-loss drugs, polycystic ovary syndrome (PCOS), pregnancy or lactation, menopause, any therapeutic medications, special dieting 6 months ago, having an acute or chronic disease such as type I and II diabetes, CVDs, hypertension, stroke, cancer, liver or renal dysfunction, and thyroid disease. Women who reported total daily energy lower than 800 kcal/d or higher than 4200 kcal/d also were also excluded [15]. The study was approved by the ethical committee at Tehran University of Medical Sciences and informed written consent was obtained from each participant. All procedures involving human subjects were approved by the Ethics Commission of Tehran University of Medical Sciences (IR.TUMS.VCR.REC.1395.1597). The research was funded by the Tehran University of Medical Sciences (Grant number: 97-03-161-41,144).

### Anthropometric and body composition assessments

Height was measured with an accuracy of 0.1 cm by a tape attached to the wall. Also, the shoulders were in a normal position, and height was measured without wearing shoes. Weight was measured to the nearest 0.1 kg with a digital scale (Seca, Germany) while participants standing and wearing a single layer of clothing. WC was measured at the umbilical site, using an outstretched tape, with no pressure to the body surface, and was recorded to the nearest 0.5 cm. Hip circumference (HC) was measured with the accuracy of 0.5 cm. Also, waist-to-hip ratio (WHR) was calculated through WC/HC. Participants’ body composition, including BMI, fat-free mass (FFM), fat-free mass index (FFMI), body fat mass (BFM) was assessed using using a bioelectrical impedance analysis (BIA) (Inbody 770 Co., Seoul, Korea [1].

### Dietary assessment, red and processed meat definition

Validated semi-quantitative food frequency questionanires (FFQ) were used for dietary assessment with 147 food items and standard serving sizes [16]. The consumption frequency of each food item or food group was classified as daily, weekly, monthly, yearly, or never [17]. The reported frequencies were converted to grams using household measurements [18]. Nutrient and energy intakes were calculated using Nutrition IV software (version 7.0; N-Squared Computing, Salem, OR). Red meat was defined as beef, veal, and sheep [19]. Processed meats were considered meats that are protected by smoking, salting, cooking, or adding chemical preservatives to the meat. Most processed meats contain beef, including red meats such as offal, sausages, burgers, and canned tuna [3, 20, 21].

### Biochemical assessment

All blood samples were collected early in the morning after a 12-hour overnight fasting. Samples were collected in tubes containing 0.1 Ethylenediaminetetraacetic acids (EDTA). Fasting plasma glucose (FPG) was assessed using an enzymatic colorimetric method with the glucose oxidase technique. TG and total cholesterol (CHOL) were measured by glycerol-3-phosphate oxidase–phenol 4-aminoantipyrine peroxidase (GPOPAP). HDL-c and low-density lipoprotein cholesterol (LDL-c) were measured by direct enzymatic clearance assays. IR was calculated by homeostasis model assessment (HOMA). HOMA-insulin resistance (HOMA-IR) was calculated as (FPG× Fasting Plasma Insulin) /22.5) [22]. All kits were from Pars Azmoon (Pars Azmoon Inc. Tehran, Iran).

### Assessment of MetS

The MetS was defined using a modification of the criteria presented by the International Diabetes Federation [23]. MetS was considered present when each participant had three or more out of the five following parameters: central obesity as WC ≥ 88 cm in women; hypertriglyceridemia as serum TG ≥ 150 mg/dL (1.69 mmol/L); HDL-c < 50 mg/dL (1.29 mmol/L); elevated blood pressure defined as SBP/DBP ≥ 130/85 mmHg; hyperglycemia as FPG ≥ 100 mg/dL (5.6 mmol/L).

### Physical activity assessment and other covariates

Physical activity (PA) was measured using the International Physical Activity Questionnaire (IPAQ) that its validity and reliability have been confirmed in the previous study [24]. This questionnaire consists of seven questions. Each question consists of two sections (number of movements per week and duration), and each section indicates participant ‘s level of physical activity (vigorous, moderate, walking, and inactive). The demographic questionnaire asked about information on age, job and marital status (single, married), educational level (undergraduate, bachelor, master degrees or higher), and economic status (low , moderate and high income) was completed by a trained nutritionist. For BP measurement, participants were at rest for at least 10 minutes and averaged to the nearest mmHg according to standard procedures on the participants’ left arm in a seated position using an automatic sphygmomanometer (OMRON, Germany).

### Statistical analysis

Data were analyzed using the Statistical Software Package IBM (SPSS Statistics version 26). Analysis of variance (ANOVA) was used to determine the difference between the means of investigated variables across means of red and processed meat group present and also reported mean and standard deviation (SD). Analysis of covariance (ANCOVA) was used to determine the difference between the means of adjusted investigated variables (age, energy intake, BMI, PA) and the reported adjusted *P*-value. To compare categorical variables, a chi-square test was used. Fisher’s exact test was used if at least 25% of cells had an expected count of less than 5. The association between higher intake of red and processed meat with MetS and its individual components was assessed by binary logistic regression. Consuming lower than the mean of red (16.32 g) and processed meat (6.48 g) was considered as a reference grouModel 1 was adjusted for age, energy intake, PA, supplement intake, and economic status. In model 2, fruits, vegetables, and dairy intake were additionally adjusted. Results were presented as odds ratios (ORs) and 95% confidence intervals (CIs) compared with the MetS and its individual components. *P*-values less than 0.05 were considered significant and *p*-value 0.05, 0.06, and 0.07 were considered as marginal significant for all tests.

## Results

### Study population characteristics

This study included 231 overweight and obese women. Clinical characteristics of participants are shown in Table 1. This was a predominantly young population, with mean age of around 36.47 years and 71.9% were married.

**Table 1:** Baseline characteristics of participants (*n* = 231)

Quantitative variables	Mean	SD
Age (year)	36.47	8.44
Weight (kg)	80.17	12.05
BMI (kg/m^2^)	30.96	4.19
WC (cm)	98.44	9.97
SBP (mmHg)	111.42	13.25
DBP (mmHg)	77.73	9.001
TG (mg/dl)	119.04	59.44
FBG (mg/dl)	87.31	9.50
HDL-c (mg/dl)	46.88	9.54
Score of red meat (g/d)	22.76	21.01
Score of processed meat (g/d)	10.82	14.62
**Qualitative variables**	**N**	**%**
**Marital status**		
Single	63	26.8
Married	168	71.9
**Education**		
Illiterate	2	0.9
Under diploma	28	12.1
Diploma	90	38.5
Master or higher degree	111	47.2
**Supplement intake**		
Yes	132	57.1
No	99	42.9

*BMI* Body mass index, *WC* Waist circumference, *HC* Hip circumference, *SBP* Systolic blood pressure, *DBP* Diastolic blood pressure, *TG* triglyceride, *FPG* Fasting plasma glucose

All data are presented as mean and SD or N and %

### General characteristics among means intake of red and processed meat

Table 2 shows univariate and adjusted population characteristics according to red and processed meat consumption. There were no differences regarding age between the groups. In univariate analysis no differences were seen on body weight, BMI, BFM, HC visceral fat area (VFA) in those consuming more red meat (*P* > 0.05), however after controlling for potential confounders, significant differences were observed (all *p* values < 0.05). The same was seen for fat mass index (FMI) in the high mean consumption of red meat after adjustment for potential confounders (*P* = 0.01).

**Table 2:** Characteristics of the study population according to consumption of red and processed meat (in g/day) in overweight and obese women (*n* = 231)

Variables	Red meat		*P*-value	*P*-value^*^	Processed meat		*P*-value	*P*- value^*^
	Low (< 16.3243 g) *N* = 196	High (> 16.3243 g) *N* = 195			Low (< 6.4888 g) *N* = 196	High (> 6.4888 g) *N* = 195		
Age (years)	37.32 ± 9.58	36.072 ± 8.79	0.18	0.50	37.52 ± 8.12	35.41 ± 8.66	**0.05**	0.26
PA (MET-minutes/week)	1025.97 ± 1473.95	1380.77 ± 2586.03	0.18	0.22	1067.564 ± 1791.97	906.010 ± 868.99	0.41	0.26
**Anthropometric variables**								
Weight(kg)	81.47 ± 12.72	80.86 ± 11.81	0.61	**0.01**	79.895 ± 11.723	80.458 ± 12.418	0.72	0.29
Height(cm)	160.76 ± 6.18	161.54 ± 5.56	0.19	0.54	160.854 ± 6.427	161.182 ± 5.544	0.67	0.62
BMI (kg/m^2^)	31.51 ± 4.37	31.03 ± 4.22	0.26	**0.01**	30.83 ± 3.94	31.08 ± 4.42	0.65	0.39
**Body composition**								
BFM (kg)	35.23 ± 9.02	34.22 ± 8.44	0.25	**0.01**	33.20 ± 7.93	34.12 ± 9.12	0.41	0.62
FFM (kg)	46.29 ± 5.75	46.71 ± 5.58	0.46	0.23	46.62 ± 5.97	46.66 ± 5.28	0.96	0.22
HC (cm)	114.66 ± 10.44	113.62 ± 9.03	0.49	**0.04**	111.92 ± 7.62	114.53 ± 10.91	0.14	0.27
WHR (cm)	1.40 ± 6.52	0.93 ± 0.05	0.30	0.10	0.92 ± 0.05	1.71 ± 8.45	0.32	0.52
SMM (kg)	25.45 ± 3.53	25.63 ± 3.31	0.59	0.21	25.56 ± 3.50	25.65 ± 3.16	0.84	0.20
SLM (kg)	43.47 ± 5.53	44.03 ± 5.26	0.31	0.44	43.97 ± 5.60	43.99 ± 4.99	0.97	0.22
VFA (cm^2^)	179.04 ± 123.89	163.36 ± 39.68	0.09	**0.04**	159.92 ± 37.66	175.38 ± 159.20	0.31	0.98
VFL	16.17 ± 3.12	17.32 ± 17.08	0.35	0.10	15.30 ± 3.36	18.24 ± 22.04	0.16	0.89
FFMI	17.88 ± 1.54	18.54 ± 9.43	0.33	0.43	17.96 ± 1.46	17.94 ± 1.42	0.91	0.09
FMI	13.73 ± 3.47	13.16 ± 3.31	0.09	**0.01**	12.91 ± 3.10	13.16 ± 3.48	0.56	0.75
**Biochemical parameters**								
CHOL (mg/dl)	184.48 ± 33.99	185.75 ± 38.30	0.78	0.33	178.74 ± 33.75	181.12 ± 30.63	0.61	0.41
TG (mg/dl)	121.33 ± 61.13	115.52 ± 58.62	0.45	0.50	116.80 ± 60.76	121.44 ± 58.25	0.59	0.54
HDL-c (mg/dl)	47.26 ± 10.40	46.39 ± 11.26	0.53	0.99	46.35 ± 10.35	47.46 ± 8.60	0.42	0.54
LDL-c (mg/dl)	95.75 ± 24.78	94.37 ± 23.72	0.65	0.36	97.13 ± 22.34	99.18 ± 23.23	0.53	0.55
OMA-IR	3.38 ± 1.30	3.30 ± 1.27	0.63	0.87	3.36 ± 1.49	3.23 ± 1.22	0.51	0.95
**Qualitative variables**								
**Marital status**								
Single	32 (48.6)	34 (51.4)	0.66	0.26	33 (51.6)	30 (48.4)	0.76	0.67
Married	84 (51.1)	81 (48.9)			83 (49.4)	85 (50.6)		
**Economic status**								
Weak	36 (65.9)	18 (34.1)	**0.002**	**0.001**	32 (59.6)	21 (40.4)	0.22	0.08
Medium	53 (47.8)	58 (52.2)			53 (45.2)	64 (54.8)		
Good	27 (41.1)	39 (58.9)			31 (50.8)	30 (49.2)		

*Abbreviations:* *PA* Physical activity, *BFM* Body fat mass, *BMI* Body mass index, *FFM* Fat-free mass, *FFMI* Fat-free mass index, *FMI* Fat mass index, *CHOL* cholesterol, *TG* triglyceride, *HC* Hip circumference, *HOMA-IR* Homeostasis model insulin resistance, *LDL-c* Low-density lipoprotein cholesterol, *SLM* Soft lean mass, *SMM* Skeletal muscle mass, *VFA* Visceral fat area, *VFL* Visceral fat level, *WHR* Waist to hip ratio

All data are presented as mean ± SD or N (%)

* *P*-value reported after adjusting total age, energy intake, BMI, and PA (BMI consider a collinear variable)

*p*-value < 0.05 is considered as a significance level and *p*-value 0.05, 0.06 and 0.07 consider as marginal significant, and they are shown in the form of bold

### Dietary food intake among means intake red and processed meat

Information about dietary intakes of the study population between the means of red and processed meat are shown in Table 3. There were significant differences in energy, carbohydrates, and total fat intakes across both means of red and processed meat (all *P* values < 0.05). As for micronutrients such as vitamin A, vitamin B1, vitamin B12, and selenium were significant in the crude model across both means of red and processed meat (*P* < 0.05), while after adjustment for energy intake, polyunsaturated fatty acids (PUFA), linoleic acid, eicosapentaenoic acid (EPA), vitamin A, vitamin B6, vitamin B5, zinc, and total fiber became significant (all *P* values < 0.05). When food groups were concernded, only vegetables consumption was significant before and after adjustment with energy intake in both means of red and processed meats (*P* < 0.05).

**Table 3:** Dietary intakes of study population between consumption of red meat and processed meat in obese and overweight women (*n* = 231)

Variables	Red meat		*P*-value	*P*-value^*^	Processed meat		*P*-value	*P*- value^*^
	Low (< 16.3243) *n* = 115	High (> 16.3243) *n* = 116			Low (< 6.4888) *n* = 115	High (> 6.4888) *n* = 116		
**Macronutrients**								
Energy intake (kcal/d)	2379.66 ± 754.65	2888.19 ± 783.82	**< 0.001**	–	2492.06 ± 726.12	2744.13 ± 698.88	**0.008**	–
Protein (g/d)	80.17 ± 27.97	102.50 ± 30.84	**< 0.001**	**0.010**	86.66 ± 28.47	91.63 ± 29.35	0.19	0.25
Carbohydrates (g/d)	337.76 ± 115.81	407.30 ± 123.65	**< 0.001**	0.33	355.18 ± 115.79	383.33 ± 113.11	**0.063**	0.21
Total fat (g/d)	86.58 ± 36.01	103.73 ± 32.17	**< 0.001**	**0.06**	88.84 ± 33.08	102.36 ± 32.48	**0.002**	0.16
CHOL(mg/dl)	224.21 ± 98.22	304.12 ± 113.19	**< 0.001**	**< 0.001**	246.28 ± 106.87	255.59 ± 109.30	0.51	0.36
SFA (g/d)	24.69 ± 9.83	32.13 ± 11.94	**< 0.001**	**0.04**	27.18 ± 11.93	29.23 ± 10.17	0.16	0.23
PUFA (g/d)	19.85 ± 11.27	20.31 ± 7.50	0.63	**< 0.001**	18.50 ± 8.07	22.60 ± 9.98	**0.001**	**0.01**
Linoleic (g/d)	17.49 ± 10.63	17.30 ± 6.99	0.84	**< 0.001**	15.74 ± 7.60	19.87 ± 9.54	**< 0.001**	**0.008**
Linolenic (g/d)	1.09 ± 0.71	1.33 ± 0.58	**< 0.001**	0.87	1.24 ± 0.69	1.25 ± 0.70	0.89	0.14
EPA (g/d)	0.02 ± 0.02	0.03 ± 0.04	**< 0.001**	**0.01**	0.03 ± 0.03	0.02 ± 0.03	0.11	**0.05**
DHA (g/d)	0.07 ± 0.81	0.12 ± 0.13	**< 0.001**	**0.02**	0.11 ± 0.12	0.09 ± 0.12	0.37	0.20
Trans fatty acid (g/d)	< 0.001 ± < 0.001	< 0.001 ± < 0.001	**0.06**	0.39	< 0.001 ± < 0.001	< 0.001 ± < 0.001	0.35	0.24
**Micronutrients**								
Vitamin A (IU/d)	647.97 ± 321.08	878.50 ± 447.86	**< 0.001**	**0.001**	795.13 ± 381.75	720.79 ± 393.77	0.14	**0.001**
Calcium (mg/d)	1143.56 ± 512.88	1394.31 ± 527.82	**< 0.001**	0.09	1148.88 ± 440.58	1182.78 ± 387.94	0.53	**0.06**
Iron (mg/d)	25.56 ± 23.52	27.30 ± 17.91	0.41	0.65	18.23 ± 5.88	19.12 ± 5.31	0.22	**0.06**
Vitamin B1 (mg/d)	1.99 ± 0.74	2.28 ± 0.69	**< 0.001**	0.11	2.01 ± 0.65	2.18 ± 0.58	**0.03**	0.89
Vitamin B6 (μg/d)	1.94 ± 0.69	2.45 ± 0.73	**< 0.001**	**0.001**	2.12 ± 0.68	2.16 ± 0.73	0.61	**0.01**
Vitamin B12 (μg/d)	3.24 ± 1.52	5.44 ± 2.75	**< 0.001**	**< 0.001**	4.03 ± 2.01	4.65 ± 2.56	**0.04**	0.50
Vitamin B5 (μg/d)	5.66 ± 1.84	7.24 ± 2.60	**< 0.001**	**0.003**	6.31 ± 2.09	6.53 ± 2.01	0.41	**0.05**
Magnesium (mg/d)	438.84 ± 171.14	512.69 ± 164.29	**< 0.001**	0.38	450.36 ± 146.87	458.15 ± 136.00	0.67	**0.001**
Zinc (g/d)	11.70 ± 4.26	15.12 ± 4.85	**< 0.001**	**< 0.001**	12.55 ± 4.21	13.16 ± 3.95	0.26	**0.04**
Selenium (μg/d)	119.54 ± 52.24	133.44 ± 45.91	**0.005**	**0.04**	113.58 ± 43.49	127.72 ± 35.59	**0.007**	0.23
Total fiber (g/d)	45.05 ± 22.53	49.64 ± 19.90	**0.03**	**0.04**	45.23 ± 17.88	44.79 ± 18.25	0.85	**0.04**
Caffeine (mg/d)	150.35 ± 170.25	156.03 ± 123.88	0.70	0.52	140.48 ± 104.88	138.60 ± 98.87	0.88	0.27
**Food groups**								
Whole grains (g/d)	7.08 ± 9.46	8.07 ± 11.26	0.41	0.86	7.96 ± 10.35	5.49 ± 7.92	**0.04**	**0.01**
Fruits (g/d)	428.37 ± 311.68	626.03 ± 335.30	**< 0.001**	**0.05**	498.55 ± 302.41	527.87 ± 351.81	0.49	0.44
Vegetables (g/d)	379.94 ± 237.16	485.40 ± 277.29	**0.001**	**0.01**	456.85 ± 262.14	380.21 ± 206.05	**0.01**	**< 0.001**
Nuts (g/d)	11.46 ± 15.61	17.17 ± 16.28	**0.002**	0.09	15.93 ± 17.35	11.80 ± 12.60	**0.04**	**0.001**
Legumes (g/d)	51.80 ± 39.07	53.55 ± 43.41	0.71	0.10	54.37 ± 44.49	52.78 ± 38.77	0.77	0.77
Tea and coffee (g/d)	728.86 ± 949.52	751.56 ± 512.78	0.79	0.96	686.70 ± 520.38	664.40 ± 483.82	0.73	0.20
Refined grains (g/d)	399.89 ± 216.99	463.71 ± 219.31	**0.01**	0.08	412.62 ± 222.25	454.99 ± 177.68	0.11	0.79
SSBs (g/d)	21.39 ± 65.31	28.57 ± 60.22	0.33	0.91	11.10 ± 20.29	34.49 ± 66.61	**< 0.001**	**0.002**
Dairy (g/d)	322.27 ± 193.67	450.85 ± 274.67	**< 0.001**	**0.04**	379.15 ± 255.51	396.77 ± 205.61	0.56	0.44
Eggs (g/d)	20.67 ± 14.01	22.66 ± 14.30	0.23	0.44	20.79 ± 12.00	21.19 ± 15.13	0.82	0.92
Fish and seafood (g/d)	8.96 ± 10.62	13.77 ± 13.08	**0.001**	0.12	10.15 ± 10.12	12.73 ± 14.72	0.12	0.25
Processed meat (g/d)	10.20 ± 13.64	11.46 ± 15.59	0.51	0.31	2.63 ± 2.04	18.95 ± 17.01	**< 0.001**	**< 0.001**
Red meat (g/d)	9.24 ± 4.29	36.34 ± 22.35	**< 0.001**	**< 0.001**	22.76 ± 19.34	22.20 ± 18.31	0.82	0.11

*Abbreviations:* *CHOL* Cholesterol, *DHA* Docosahexaenoic acid, *EPA* Eicosapentaenoic acid, *PUFA* Polyunsaturated fatty acid, *SFA* Saturated fatty acid, *SSBs* Sugar-sweetened beverages

All data are presented as mean ± SD

*P*-values result from the ANOVA test

* *P*-value reported after adjusting energy intake with ANCOVA test

*p*-value< 0.05 is considered as a significance level and *p*-value 0.05, 0.06 and 0.07 considered as marginal significant, and they are shown in the form of bold

### Association of red and processed meat intake with MetS

Table 4 shows the univariate and adjusted associations between red and processed meat intake with MetS and its components. In binary logistic regression analysis in the crude model, higher intake of red meat (OR:1.13, 95% CI:0.58,2.20, P:0.70) and processed meat (OR:1.23, 95% CI:0.54,2.80, P:0.21) were not associated with the MetS. After controlling for potential confounders such as fruits, vegetables, and dairy intake in model 2, there was a marginal significant association between higher intake of processed meat and increasing the odds of MetS (OR:1.01, 95% CI:0.94,2.94, P:0.06).

**Table 4:** Association between red meat and processed meat intake with MetS and its components in obese and overweight women (*n* = 231)

Variables		Red meat		*p*-value	Processed meat		*p*-value
		OR	CI (95%)		OR	CI (95%)	
MetS	Crude	1.13	0.58,2.20	0.70	1.23	0.54,2.80	0.21
	Model1	1.15	0.40,3.29	0.78	1.10	0.91,2.94	0.98
	Model2	1.39	0.42,4.60	0.58	1.01	0.94,2.94	**0.06**
High WC (cm)	Crude	0.53	0.27,1.04	**0.06**	0.88	0.38,2.04	0.78
	Model1	0.32	0.11,0.96	**0.04**	0.89	0.33,2.37	0.82
	Model2	0.31	0.10,0.97	**0.04**	0.82	0.28,2.40	0.72
High TG (mg/dl)	Crude	0.93	0.51,1.70	0.81	1.47	0.74,2.92	0.26
	Model1	0.92	0.37,2.26	0.86	1.49	0.66,3.33	0.12
	Model2	0.82	0.33,2.06	0.68	1.27	0.94,2.98	**0.07**
Higher than median of HDL-c (mg/dl)	Crude	1.25	0.74,2.10	0.40	0.68	0.37,1.24	0.21
	Model1	1.33	0.61,2.89	0.46	0.59	0.29,1.21	0.15
	Model2	1.31	0.59,2.88	0.50	0.64	0.30,0.95	**0.03**
High FPG (mg/dL)	Crude	0.55	0.20,1.47	0.23	0.78	0.26,2.35	0.66
	Model1	0.23	0.04,1.17	**0.07**	0.46	0.11,1.85	0.27
	Model2	0.25	0.04,1.35	0.10	0.31	0.06,1.53	0.15
High SBP (mm-Hg)	Crude	1.24	0.57,2.68	0.58	0.58	0.23,1.45	0.24
	Model1	0.95	0.27,3.27	0.93	0.61	0.19,1.96	0.41
	Model2	1.08	0.30,3.87	0.89	0.61	0.17,2.14	0.44
High DBP (mm-Hg)	Crude	1.07	0.59,1.92	0.81	1.13	0.58,2.19	0.71
	Model1	1.02	0.44,2.34	0.96	1.10	0.50,2.41	0.79
	Model2	1.14	0.46,2.82	0.77	0.99	0.43,2.26	0.98

*Abbreviations:* *CI* Confidence interval, *DBP* Diastolic blood pressure, *FPG* Fasting plasma glucose, *HDL-c* High-density lipoprotein cholesterol, *MetS* Metabolic syndrome, *OR* Odds ratio, *SBP* Systolic blood pressure, *TG* Triglyceride, *WC* Waist circumference

Model 1: adjusted by age, energy intake, PA, supplement intake, economic status

Model 2: adjusted by model 1 further with fruits, vegetables, and dairy intake

Receiving lower than median of red meat (16.3243) and processed meat (6.4888) is considered as a reference group

*p*-value < 0.05 is considered as a significance level and *p*-value 0.05, 0.06 and 0.07 consider as marginal significant, and they are shown in the form of bold

### Association of red and processed meat intake with higher WC

Table 4 also shows that in the crude model, there was a marginally significant association between higher intakes of red meat and lower odds of higher WC that after adjustments became significant, in both models 1 and 2.

### Association of red and processed meat intake with higher TG

After adjustment in model 2, there was a marginally significant association between higher intakes of processed meat and the odds of higher TG (OR:1.27, 95% CI:0.94,2.98, P:0.07).

### Association of red and processed meat intake with lower HDL-c

After adjustment in model 2, there was significant association between higher intakes of processed meat was associated with greater odds of having lower HDL-c (OR:0.64, 95% CI:0.30,0.95, P:0.03) (Table 4).

### Association of red and processed meat intake with higher FPG

In crude and adjusted models no observed significant associations were encountered between a higher intake of processed meat and odds of higher FPG. Furthermore, after adjusting in model 1, a marginally significant association between higher intakes of red meat and lower odds of higher FPG (OR:0.23, 95% CI:0.04,1.17, P:0.07) was seen (Table 4).

### Association of red and processed meat intake with higher BP

There were no significant associations between higher intakes of red or processed meat and odds for increased BP in both crude and adjusted models (*P* > 0.05).

## Discussion

The present cross-sectional study assessed the association between consumption of red and processed meats and MetS and its components in healthy obese and overweight Iranian women. There were marginally significant associations between higher intake of processed meat and odds of MetS presence. Of importance, there was a significant associations between high intake of red meats with lower odds of higher. There was a significant associations were found between high intake of processed meats with greater odds of having lower HDL-c.

There is previous evidence associating high intake of processed meat with MetS in women [3]. Kim et al. found that in the high category of processed meat intake compared to the low category, the risk of MetS increased by about 35%. Several mechanisms have been proposed to explain this association as high intake of CHOL, iron, SFAs, nitrites, and nitrates found in meat and metabolic disorders [12, 25, 26]. Moreover, excess adiposity, elevated oxidative stress induced by heme iron, the deleterious effects of the known preservatives that are routinely added to processed on pancreatic beta cells may cause hyperinsulinemia and ensuing insulin resistance [3, 8]. More than that, the associated low-grade systemic inflammatory state in people with higher intakes of processed meat, [27], has also been implicated on the association of processed meat, but not red meat, on increasing the risk of MetS.

On this study we observed a significant inverse association between greater intake of red meat with lower odds of higher WC values, this finding is probably due to lower consumption than the average consumption of other populations. A cross-sectional study on older Australian women found that a red meat-limited diet is significantly associated with lower BMI, body weight, and WC [28]. Several studies show that high consumption of red meat is significantly associated with increased WC and weight gain [29–31]. The supportive pieces of evidence mention that this hypothesis that high intakes of red meat could increase WC, could be due to being rich in CHOL and SFAs and following high energy density, and its effects on weight gain, adiposity, and higher WC [32]. Also, another new investigation conducted by Mazidi et al. in the USA on a representative sample of adults, suggests that a higher intake of SFAs, in particular, can raise the amount of white adipose tissues by activating several inflammatory responses in the consumer’s body. These researchers also found that the raising iron load in the liver is strongly associated with an interruption in insulin/glucose function, elevates the plasma levels of blood glucose by adverse effects on glucose liver production, and contracts to reuptake of glucose due to inducing insulin resistance [33].

We also observed a significant association between a higher intake of processed meat and greater odds of having lower HDL-c, and also a marginally potential correlation with higher levels of TG, but we found no significant results about the relationship between red meat and lipid profile components. Results of the current study have been supported by the longitudinal studies of Simpson et al. in 2019 that found reducing processed meat intake from 1.3 to 0.7 portions a day can increase HDL-c and reduce LDL-c levels in adults [34]. Also, Leffa et al. showed that processed food, in particular, processed meat can significantly raise TG and total CHOL concentrations in children [35]. In addition, a cohort study on Korean adults also showed high consumption of red and processed meat could increase the risk of dyslipidemia in this population [36]. In this regard, a cohort study with 4071 Swedish adults, showed that the Western dietary pattern associated with a higher intake of red and processed meat can associated with lower levels of HDL-c, and increased levels of TG [34]. But a recent meta-analysis on 36 RCTs in 2019 found the opposite result [37]. Red and processed meats are the main sources of trans fatty acids intake in most populations [38]. As we know from current references, trans-fatty acids play a role in the metabolism of lipoproteins, and significantly decrease HDL-c, and increase LDL-c and TG concentrations [39]. In vitro, this type of fat is associated with a change in the secretion, composition, size of apolipoprotein B-100 and in humans with a decrease in the size of LDL-c [40–42]. Cholesteryl ester transfer protein (CETP) plays in reverse cholesterol transport. With raising of TG levels, CETP transfer of cholesteryl esters to very low-density lipoproteins (VLDL) [43]. CETP inhibitor causes an increase in HDL-c levels [44].

This study has some limitations. First, dietary data were collected by FFQ, which misclassification of dietary intakes is considered an inherent limitation. Second, it was difficult to determine causal relationships in this cross-sectional study, and even after controlling the confounders, there may be residual confounding. Third, due to working on specific sex and overweight and obese people, this result cannot be generalized to men and people of other weights. More data are therefore necessary to better clarify the causal role of red and processed meat on MetS and its components inlcuing lon-term randomized trials.

## Conclusions

Our study suggests that a higher consumption of processed meats is associated with a higher risk for presence the MetS and its components in Iranian women with excess adiposity. However, this may not be the case for red meat.

## Data Availability

Authors declare that the data of this study are provided in this article, and all the data in the study will be available with the opinion of the corresponding author.

## Abbreviations

BIA: Bioelectrical impedance analysis
BFM: Body fat mass
BMI: Body mass index
BP: Blood pressure
CHOL: Cholesterol
CI: Confidence interval
CETP: Cholesteryl ester transfer protein
CVDs: Cardiovascular diseases
DBP: Diastolic blood pressure
DHA: Docosahexaenoic acid
EDTA: Ethylenediaminetetraacetic acid
EPA: Eicosapentaenoic acid
FFM: Fat-free mass
FFMI: Fat-free mass index
FMI: Fat mass index
FFQ: Food frequency questionnaire
FPG: Fasting plasma glucose
GPOPAP: Glycerol-3-phosphate oxidase–phenol 4-aminoantipyrine peroxidase
HDL-c: High-density lipoprotein cholesterol
HCAs: Heterocyclic amines
HC: Hip circumference
HOMA: Homeostasis model insulin resistance index
IR: Insulin resistance
IPAQ: International physical activity questionnaire-short form
LDL-c: Low-density lipoprotein cholesterol
MetS: Metabolic syndrome
MUFA: Monounsaturated fatty acid
OR: Odds ratio
PCOS: Polycystic ovary syndrome
PA: Physical activity
PUFA: Polyunsaturated fatty acid
RCT: Randomized controlled trial
SBP: Systolic blood pressure
SD: Standard deviation
SFA: Saturated fatty acid
SLM: Soft lean mass
SMM: Skeletal muscle mass
SSBs: Sugar-sweetened beverages
VFA: Visceral fat area
TG: Triglyceride
TLGS: Tehran Lipid and Glucose Study
TUMS: Tehran University of Medical Sciences
VFL: Visceral fat level
VLDL: Very low-density lipoproteins
WC: Waist circumference
WHR: Waist-Hip ratio
